# How Should Ongoing Surgical Care Needs be Met During Global Health Engagements?

**DOI:** 10.1111/dewb.70041

**Published:** 2026-05-22

**Authors:** John S. Oh, Tamara J. Worlton, Carolyn Gosztyla

**Affiliations:** ^1^ Department of Surgery Milton S. Hershey Medical Center Hershey PA USA; ^2^ Department of Surgery Uniformed Services University of the Health Sciences Bethesda MD USA

**Keywords:** bio‐ethics, global health engagement, global surgery

## Abstract

The Department of Defense has a long history of Global Health Engagement, beginning with identifying and developing vaccines for yellow fever, influenzae, and malaria. Contemporary examples of global health engagement include the worldwide deployment of military assets to aid in natural disasters, and deployment of ship and land based medical teams for short‐term medical and surgical engagements. In 2017, the Department of Defense Instruction on Global Health Engagement was published to help guide these efforts. Surgery is an indispensable part of global health and presents unique ethical challenges.

## 1 Vignette

Dr. J is an active‐duty general surgeon on a forward surgical team deployed to Central Asia. The surgeon's post residency career was spent in a military community hospital that predominantly does outpatient surgery. The surgical team consists of an operating room technician, an operating room nurse, a licensed practical nurse, and two medics. They have been in country for several months and have not performed any complex operative cases during their deployment.

Base personnel often encounter local national civilians in need of medical assistance. They are brought onto the base to be treated by the surgical team. These engagements are viewed favorably by both the local community and the base commander. On one mission, a local adult male with a groin hernia presents to the surgery team. The hernia been present for several years and contains a large amount of bowel. While the bowel is not obstructed, it causes pain with straining. The surgical team is unaware of the local national medical capabilities, nor have they met with any local physicians. The patient is eager to have an operation to fix the hernia as it keeps him from working and affects his quality of life.

The surgeon weighs the risks of operating as large hernias have a relatively high complication rate. The surgeon elects to perform the operation, which is completed successfully. However, the patient was discharged from the medical facility earlier than anticipated. There was a rocket attack on the base, and all further medical care of local nationals was halted, including follow‐up care. The surgical team returned home a few months later, and the patient was not seen again.

## 2 Commentary

Cases like the one above illustrate how military deployments to conflict zones place medical providers in situations where they are required to navigate ethical issues. During the recent conflicts in Afghanistan and Iraq, trauma care was the prime focus of the Department of Defense (DoD). Other than a cursory pre‐deployment brief on the first Geneva Convention, there was no pre‐deployment training regarding the role of DoD surgical assets in civilian care. These engagements were often taken on under the umbrella of humanitarian care.

A review of 11 years of humanitarian surgical care at U.S. military treatment facilities in Afghanistan highlighted a need to address ethical issues that arise during civilian aid. The issues highlighted in the article included the lack of sustainability, complication management, and conflicts with local national healthcare providers [1]. The following is a commentary on the evolution of DoD global health engagement and potential ethical conflicts that may arise with a focus on surgical care. These include competing loyalties between military objectives and patient care, outcomes and sustainability of surgery in conflict zones, and the surgical training requirements of the DoD.

## 3 Competing Loyalties

The military surgeon involved with GHE is faced with many ethical issues [2]. At the crux of them is the issue of dual loyalty, where the military surgeon has their oath to the patient but also their oath to the military. While in principle these oaths are not contradictory in GHE, in practice they can be.

In conflict zones, the DoD utilized medical engagements to help bolster support among the local population. The best documented programs include Medical Civic Action Programs (MEDCAP) during the Vietnam War [3]. These missions were the first documented in the “winning hearts and minds” doctrine. Although there was little reported positive outcome from these engagements, they were adopted again in the Iraq and Afghanistan conflicts. While rewarding on an individual level, post hoc analysis showed unintended consequences of these engagements. These included contributing to “brain drain”, creating dependence on the US, and the lack of coordination with and encroachment in other humanitarian efforts [4].

To address these issues, the DoD published the DoD Instructions on GHE in 2017 [5]. This document provides guidance for GHE activities with partner nations. It includes the promotion and enhancement of the partner nation's stability and security. However, the explicit core focus of the DoD Instruction (DoDI) is to support U.S. government national security objectives. The DoDI also explicitly outlines that the policy seeks to “enhance the readiness of DoD medical forces”. GHE is thus outlined as a “tool” to achieve those objectives. Contemporary examples of GHE missions include ship‐based operations staged from the USNS Comfort, USNS Mercy, such as Continuing Promise and Pacific Partnership and the US Air Force Pacific Angel. Short term missions such as surgical readiness training exercise (SURGRETE) are additional examples of GHE utilized by the DoD.

## 4 Sustainability

To mitigate risks during limited engagements, teams will do “low risk” surgeries, but these types of operations are often readily available in the community. Assistance with complex cases is what is usually needed. Additionally, procedures that would typically be considered “low risk” in the US healthcare system may have a higher risk profile in low‐ and middle‐income countries (LMIC). The LMIC may not have parallel resources (CT scans, surgical equipment, medications) to diagnose and treat complications in the same time frame as in the US. This leaves patients vulnerable to surgical complications after DoD surgical resources are withdrawn.

Many countries prefer engagements that help with ongoing programs, such as reconstructive surgery for complex orthopedic injuries or transplant surgery. However, short‐term funding and constantly rotating personnel in the DoD make efforts like these difficult. Furthermore, quickly executed missions lead to planning that does not involve local surgeons, does not account for local needs, and does not have explicit follow up. For the DoD surgical personnel, seeing the need in the local community but being unable to help also creates moral injury.

Building sustainable surgical capacity can also be in the direct interests of the DoD and national security. Increasing surgical capacity improves a nation's overall healthcare capability by enhancing the ability to respond to natural disasters and pandemics. During the COVID 19 pandemic, operating rooms and post‐operative care units were used to house critically ill patients. Furthermore, anesthesiologists and post operative care staff were leveraged to manage COVID 19 patients [6]. Robust and resilient medical systems saved lives by providing valuable surge capacity [7]. As LMICs are most susceptible to governmental destabilization during epidemics, mitigating the consequences of a pandemic can prevent economic devastation and political instability [8]. This is of vital interest to the US and global national security.

## 5 Surgical Training Requirements in the Dod

The U.S. Defense Health Agency also recognizes that critical wartime skills are essential during times of conflict [9]. Decreasing operative volume and complexity in surgical cases at military treatment facilities led to innovative solutions to maintain surgical readiness within the DoD. One solution has been the establishment of military‐civilian partnerships (MCPs) which embed military teams into civilian trauma centers within the US. However, the modern level one trauma center in the US is not as resource constrained nor austere as a deployed environment.

Another proposed solution is collaborating with LMICs. One example of this is the Joint Task Force Bravo mission in Honduras. This program partners with local hospitals and has hosted decades of rotating teams of military physicians [10]. The issue of follow‐up and coordination with local physicians is well addressed, but there are nuanced considerations. This mission has been ongoing for almost 30 years, but we are left with the following questions: Are we creating dependency without addressing capacity building? What would happen if this mission and its funding abruptly ceased? The large trauma volume in a low‐resource setting makes excellent training for military doctors, but what is our responsibility as physicians to increase local resources or provide injury prevention programs?

GHE should aim to deliver equitable surgical care worldwide through partnerships with LMICs. These partnerships need to be carefully designed with the LMIC to foster and develop bilateral capabilities in training, research, and education. The goal is to improve both the quality and the quantity of surgical care [11]. This requires understanding and acceptance of the LMICs priorities.

## 6 Conclusion

In conclusion, global surgical engagements should not be structured as a one‐sided affair and careful consideration for reciprocal benefit with a focus on capacity building is paramount for success. Improvement of global health can result in the prosperity of the world. Enhanced prosperity will result in international stability and security, which are of vital interest to the US [12].

## Disclosure

The opinions or assertions contained herein are the private ones of the author/speaker and are not to be construed as official or reflecting the views of the Department of Defense, the Uniformed Services University of the Health Sciences or any other agency of the U.S. Government.

The views expressed in this article reflect the results of research conducted by the author(s) and do not necessarily reflect the official policy or position of the Department of the Navy, Department of Defense, nor the U.S. Government.

## Conflicts of Interest

The authors declare no conflicts of interest.

## Data Availability

Data sharing not applicable to this article as no datasets were generated or analysed during the current study.
